# Case Report: Treatment of Open Femoral Shaft Fracture in a Severely Burned Patient

**Published:** 2008-03-26

**Authors:** Tai-Li Chang, Robert J. Spence, Simon C. Mears

**Affiliations:** Department of Orthopaedic Surgery, Johns Hopkins University/Johns Hopkins Bayview Medical Center, Baltimore, MD; Division of Burn Surgery, Johns Hopkins University/Johns Hopkins Bayview Medical Center, Baltimore, MD

## Abstract

**Objective:** To present a case report of a patient with an open fracture and severe burns and review the literature. **Methods:** The patient was treated with intubation, intravenous antibiotics, and debridement and intramedullary nailing for the femur fracture. He later underwent multiple burn excision procedures with allograft and autograft skin coverage. The wound over the fracture was treated with dressing changes. The fracture was treated with nail exchange and bone grafting for atrophic nonunion. **Results:** The patient was returned to full weightbearing and good function with a fully healed femur. **Conclusions:** Treatment of open fractures in burn patients should be tailored to the specific needs of the individual; they should be reduced and stabilized via internal fixation at the earliest opportunity and should be managed by minimizing wound colonization through successive debridement, wound care, and consideration of flap coverage.

Orthopedic care of fractures in burn patients has evolved from a conservative to a more aggressive approach over the past 50 years. Casting, splinting, traction, and external fixation were the initial standards of care.^1^–^4^ Fracture reduction and stabilization through internal fixation is an approach that has been popularized within the past decade.^5^ No standard exists for the orthopedic management of open fractures complicated by burns. Also, there are only limited numbers of published case reports of open fractures associated with burns.^6^–^9^ The location of burns and the medically compromised nature of the severely burned patient may make the standard reconstructive ladder of local, regional, and free-flap coverage impossible to follow. We present a patient with a 40% total body surface area, deep dermal and full-thickness (“third-degree”) burn, and a type IIIB^10^,^11^ open midshaft femur fracture that was treated with initial debridement and intramedullary nailing. Wound closure was achieved with local dressing changes. Femoral nonunion was later treated with nail exchange and bone grafting to gain fracture union.

## CASE REPORT

**Figure 1:**
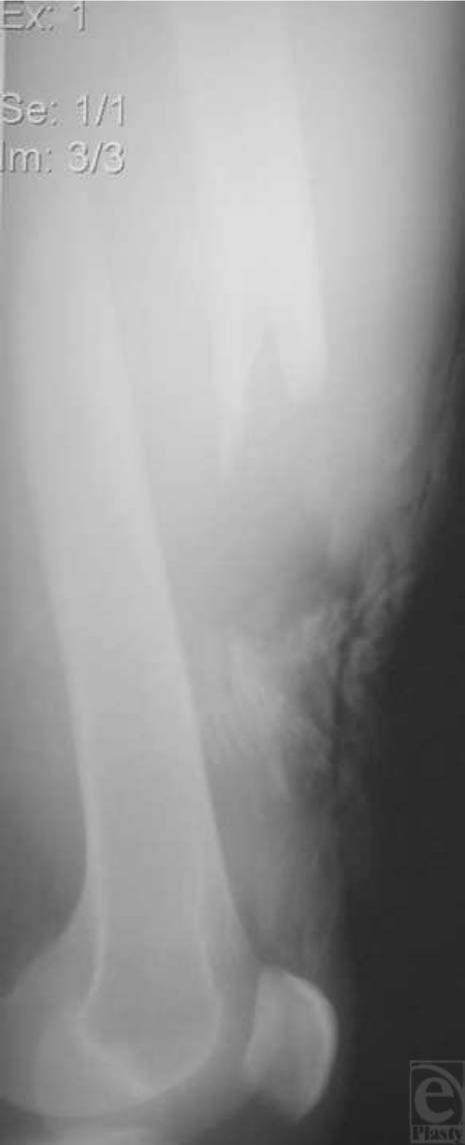
Lateral radiograph of the right femur shows a type IIIB open fracture. Note the soft tissue burn injuries adjacent to the fracture.

In August 2003, a 43-year-old man presented to the emergency department with severe burns and a grossly deformed and bleeding right thigh after a motorcycle collision. He had become trapped under the motorcycle, which caught on fire. The patient was intubated after arrival for smoke inhalation injury. Physical examination revealed a small puncture wound laterally and 2 puncture wounds anteriorly in the midthigh, the larger of which was approximately 5 mm in diameter. The patient had a 40% total body surface area burn, all of which was deep dermal and full-thickness (“third-degree”) burn with the exception of his face, which was superficial. The fracture was within the burned area. Palpable pulses were present distally, but motor and sensory examinations could not be obtained. Anteroposterior and lateral (Fig 1) radiographs revealed a right femoral shaft fracture.

**Figure 2:**
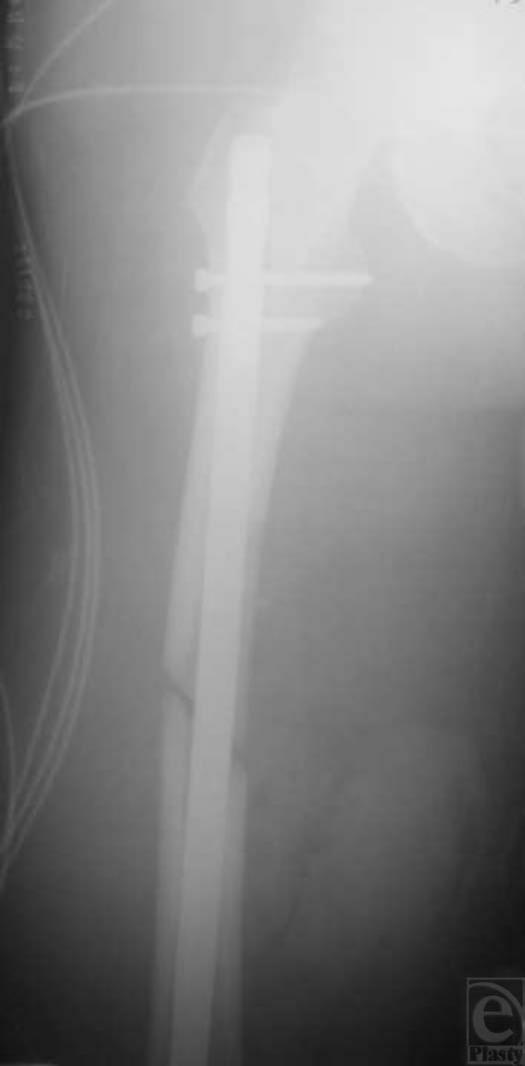
Postoperative anteroposterior radiograph shows reduction and intramedullary fixation of the right femur.

The patient received intravenous cefazolin and a tetanus immunization in the emergency department and was admitted to the burn intensive care unit. The open femur fracture was irrigated with a liter of isotonic sodium chloride solution. The wounds were dressed, and the leg was splinted. The patient was taken to the operating room for debridement of the fracture and skeletal stabilization. The nonviable skin edges of the open fracture sites were debrided sharply. Gross contamination, which included several pieces of grass or other organic material, was removed. The patient's open fracture was then irrigated with 10 L of isotonic sodium chloride solution via pulse lavage. After the open fracture had been irrigated thoroughly, instruments were changed, and attention was turned to stabilizing the femur. The patient was transferred to a fracture table. After fracture reduction, the femoral canal was reamed to 15 mm, and a 14 × 380 mm locked T2 femoral nail (Stryker, Mahwah, NJ) was placed (Fig 2). The rotational alignment and length of the patient's right leg were checked in comparison to the left leg and thought to be acceptable. The open fracture wounds were closed loosely with 2-0 vertical mattress nylon sutures. The patient was kept intubated and was transferred to the burn intensive care unit.

The patient had repeated episodes of sepsis with gram-negative organisms resistant to multiple antibiotics. He remained intubated because of his inhalation injury. He was treated with multiple burn excision procedures with allograft and, subsequently, autograft skin coverage. He had sustained burns to the eyes and developed a *Pseudomonas* infection that was managed by ophthalmology.

Ten days after admission, a percutaneous tracheotomy was performed. Twelve days after presentation, the open fracture wounds began to drain. The medial thigh wound was redebrided in the operating room with removal of a fragment of nonviable bone. The wound could not be closed and was packed with chlorpactin (Clorpactin WCS-90, USA Guardian Laboratories, Hauppauge, NY) wet-to-dry dressings. The patient remained critically ill and was not a candidate for flap closure. A vacuum-assisted closure (VAC) device was not used because of the surrounding burns to the thigh. The patient underwent a total of 10 burn excision and skin grafting procedures to close his burn wounds. Dressing changes to the thigh were continued while the patient remained intubated. He was not thought to be a candidate for local flap or free-flap treatment because of his gram-negative sepsis and the area of surrounding damage to the thigh. Granulation tissue was seen at the base of the thigh wound, and it began to close by secondary intention. The patient was weaned from the ventilator and extubated 2 months after admission. He was mobilized and transferred to a rehabilitation unit 10 weeks after injury.

**Figure 3:**
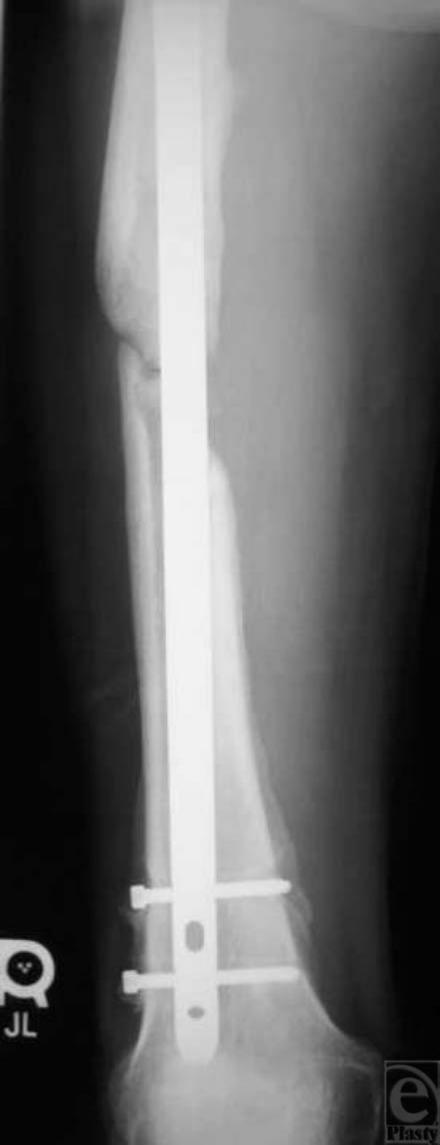
Anteroposterior radiograph approximately 1 year after injury shows atrophic nonunion of the fracture.

**Figure 4:**
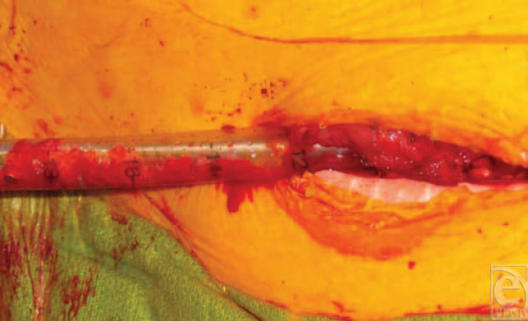
Intraoperative photograph of cancellous bone chips packed into the femur through a chest tube.

**Figure 5:**
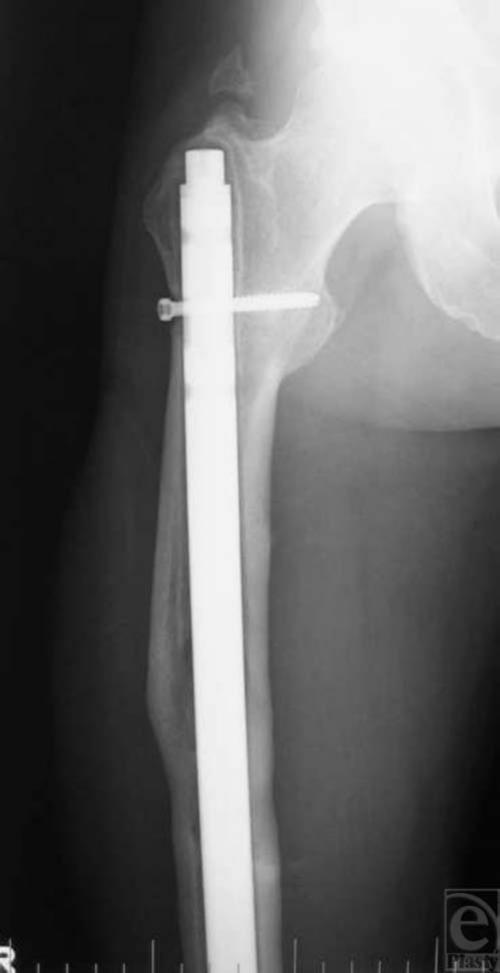
Radiograph showing final healing of the femoral shaft fracture.

**Figure 6:**
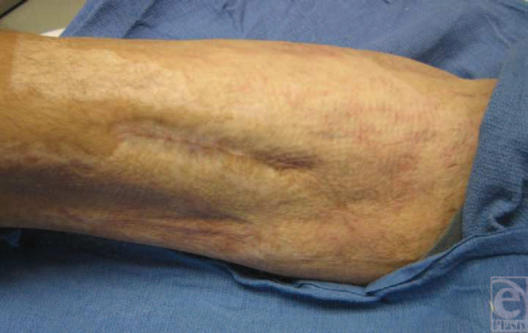
Healed medial thigh wound 3 years after the injury.

Approximately 1 year later, the patient returned to the orthopedic clinic with a painful atrophic nonunion of his fracture (Fig 3). The nail appeared to be loosening, so dynamization was not considered to be an option. His thigh wounds had healed completely. The nonunion was treated with reaming, allograft cancellous chips (impacted through the medullary canal via a chest tube before nailing), and nail exchange (a larger femoral nail) (Fig 4). In 3 months, the patient was able to ambulate and bear weight on his right leg without pain. Two and a half years after the index procedure, the patient's painful distal locking femoral screws were removed. The patient proceeded to return to full weightbearing and is functioning well with a fully healed femur (Fig 5). Full soft tissue coverage and healing of the thigh wound were achieved (Fig 6).

## DISCUSSION

The goal of orthopedic intervention in patients with burns is to obtain fracture stabilization via the earliest possible fracture reduction and concurrently provide for optimal wound care and early patient mobility. Fracture stabilization allows for increased ventilation, improves pain control, and facilitates transfers for dressing changes. Internal fixation is best done when wound colonization is lowest, which minimizes the risk of infectious complications.^12^ Damage control orthopedics with initial external fixation of fractures should be considered, especially in the presence of multiple fractures.^13^

The standard of orthopedic care for patients with burns and fractures has changed with time. With regard to early experience, reports in the 1940s showed that during World War II, compression dressings and cylinder casts or skeletal traction were used to avoid pin placement through burned tissues.^1^,^14^ In the 1960s, Grisolia et al^152^ showed that in dogs with femur fractures and clean wounds, 93% of the fractures treated via open reduction internal fixation through overlying full-thickness burns healed. Grisolia and Peltier^16^ later found that only 86% of fractures in clean wounds healed and 93% of fractures in infected cases progressed to a nonunion if the burn wounds were excised and closed before fracture reduction. Conversely, Dowling et al^17^ showed that internal fixation of fractures in patients with burn was contraindicated until the burn had healed. They thought that the burn wounds seeded the bones with bacteria. Reports in the 1970s championed other conservative orthopedic interventions for burn patients with fractures, such as skeletal traction (seen as the preferred method of stabilization because it permitted ease of inspection and application of antimicrobials^18^) and external fixation and plaster casting.^2^,^6^

In recent decades, more aggressive orthopedic interventions have been shown to be successful in the treatment of fractures in burn patients. Saffle et al^5^ showed excellent results through internal fixation in burn patients and indicated that such orthopedic procedures must be performed within 48 hours of injury if the incision is made through or adjacent to the burn. In a study of 101 patients treated for major fractures and burn injuries during a 10-year period, Dossett et al^12^ found that rigid internal fixation of fractures was associated with fewer orthopedic complications and it can be safely performed within the first 48 hours. They recommended that the timing of the orthopedic intervention should be determined *a priori* by the patient's clinical and hemodynamic status, the length of orthopedic procedure, and the expected blood loss. They also indicated (although details were not reported) that attempts to salvage limbs with concomitant fractures and burn injuries of extremely large magnitude might be imprudent. Fracture immobilization or skeletal traction was recommended if the patient's clinical or hemodynamic status prohibited internal fixation. They reported only 1 nonunion (of a femoral neck fracture) and no other late complications related to internal fixation of any fracture.

Despite numerous publications on the orthopedic management of patients with burns with fractures, to our knowledge, there are only 6 reports that focus specifically on the management of burn patients with open fractures.^7^–^9^,^12^,^19^,^20^ Four of those studies were case reports published before 1984^7^,^8^,^19^,^20^ and 3 of those were published in languages other than English.^7^,^8^,^20^ In 1984, Wang et al^9^ reported on a patient who had a 95% total body surface area, third-degree burn with an open comminuted fracture of the tibia, and fibula that was treated with manipulation and a plaster cast. In the series reported by Dossett et al,^12^ 4 patients had open fractures, which were treated with intramedullary nailing or open plating. The authors advocated that all open fractures must have debridement within 24 hours of injury.

Our patient was treated with acute debridement and intramedullary nailing with a reamed nail. Because of the large size of the patient's canal, we used a 14-mm nail. Controversy exists about the timing of nailing in the acutely injured patient and about the use of an unreamed or reamed nail.^21^ It has been suggested by Pape and colleagues^22^ that acutely injured patients, especially those with lung injury, should be treated with initial external fixation and delayed nailing. Other investigators,^23^ however, have reported no differences in pulmonary outcomes with acute nailing. We thought that our patient was medically stable enough for nailing and this procedure would avoid the placement of external fixation pins through burned areas of skin. Our patient had several risk factors for nonunion, including a small gap at the time of initial nailing, substantial bone loss at the fracture site, and an open injury.^24^ Although it is debated, some authors^25^,^26^ have suggested that a larger reamed nail is helpful in preventing nonunion. Pape and Giannoudis^27^ have shown evidence of thermal injury and fat embolism with the use of reamed nails. We used a large reamed nail in our patient in an unsuccessful attempt to prevent nonunion. Additional study is required to determine whether reaming is indicated or contraindicated in patients with burns at the site of open fractures.

Current experience with respect to orthopedic intervention in this patient population is anecdotal. To our knowledge, there are no published reports that have randomized patients or a control group for the comparison of orthopedic intervention outcomes. Until such a study is published, we believe that treatment of open fractures in burn patients should be tailored to the specific needs of the individual; they should be reduced and stabilized via internal fixation at the earliest opportunity and should be managed by minimizing wound colonization through successive debridement, preferably with the first debridement within the first 24 hours. If possible, consideration for wound closure should follow the “revised” plastic surgery reconstructive ladder, in which VAC is on the same step as a free flap.^28^ Free-flap coverage should be considered in wounds with open bone or joint exposure. Severely burned patients may not be candidates for flap coverage, and for these patients, we recommend local dressing changes or VAC for wound management. VAC has been shown to decrease excess fluid, decrease bacterial count, improve microcirculation, and eliminate factors (such as cytokines and collagenases) that inhibit wound healing.^29^–^33^ The choice of using local dressings or VAC should be based on the condition of the skin around the wound. The VAC must be able to achieve suction, which may not be possible with severe adjacent skin damage. We also recommend routine follow-up to assess for fracture nonunion because reoperation may be needed to ensure a successful recovery for a burn patient with an open fracture.
